# Measuring the efficiency of treatment with neurofeedback method in patients with Affective disorders

**DOI:** 10.1192/j.eurpsy.2025.1362

**Published:** 2025-08-26

**Authors:** A. Risteski, S. Arsova, S. Mitrovska

**Affiliations:** 1 Department of Affective Disorders; 2 Day Hospital, University Clinic of Psychiatry, Skopje, North Macedonia

## Abstract

**Introduction:**

Affective disorders are in constant rise and they are a serious medical and social problem. All the latest studies suggest that the incidence of affective disorders is about 14%, which makes them one of the most common psychiatric disorders. Because of that, there is a need for alternative forms of treatment of affective disorders.

**Objectives:**

The main goal of this study is to evaluate the effectiveness of the combined treatment of pharmacotherapy together with the Neurofeedback method in the treatment of affective disorders in adult individuals.

**Methods:**

A prospective randomized study including a total of 100 outpatients from University Clinic of Psychiatry in Skopje. The subjects were randomly divided into 2 groups of 50 patients, of which the patients in the study group were treated with a combined treatment that includes the neurofeedback method as an augmentation to antidepressant therapy, while the control group with monotherapy with antidepressant medications. Subjects were examined for 2 months, with evaluation of despressive symptoms 2 weeks after the start of treatment, and then after 1 month, using the following psychodiagnostic instruments: psychiatric interview; short non-standardized socio-demographic questionnaire, CGI, HAMD, HAMA and Beck depression scale.

**Results:**

Patients in the study group, treated with a combined approach of antidepressant therapy and the neurofeedback method, showed a significantly greater reduction in symptoms of depression and anxiety compared to patients in the control group treated with antidepressants as monotherapy. According to the HAMA anxiety scale, the mean score in the study group (SG) decreased by 2.86 points (p<0.0001), compared to a decrease of 0.84 points in the control group (CG) (p=0.0017). On the Beck Depression Scale, the mean score in the decreased by 2.08 points (p<0.0001), compared to a decrease of 0.44 points in the CG (p=0.022). On the HAMD depression scale, the average score in the study group (SG) decreased by 2.42 points (p<0.0001), while in the control group (CG) the decrease was 0.12 points (p=0.63), the CGI scale showed a significant reduction in global symptoms in (SG) (p=0.0035), which further confirms the positive effects of the combined treatment.

**Conclusions:**

The research shows that neurofeedback treatment in addition to pharamcological therapy leads to significant improvement in depressive and anxiety symptoms.

**Disclosure of Interest:**

None Declared

